# Daily oviposition patterns of the African malaria mosquito *Anopheles gambiae *Giles (Diptera: Culicidae) on different types of aqueous substrates

**DOI:** 10.1186/1740-3391-2-6

**Published:** 2004-12-13

**Authors:** Leunita A Sumba, Kenneth Okoth, Arop L Deng, John Githure, Bart GJ Knols, John C Beier, Ahmed Hassanali

**Affiliations:** 1 International Centre of Insect Physiology and Ecology (ICIPE), PO Box 30772, Nairobi, Kenya; 2 Department of Zoology, Egerton University, PO Box 536, Njoro, Kenya; 3 Entomology Unit, Agency's laboratories Seibersdorf, International Atomic Energy Agency, A-1400, Vienna, Austria; 4 University of Miami School of Medicine, Department of Epidemiology and Public Health. Highland Professional Building, 1801 NW 9th Ave., Suite 300 (D-93), Miami, FL 33136, USA

## Abstract

**Background:**

*Anopheles gambiae *Giles is the most important vector of human malaria in sub-Saharan Africa. Knowledge of the factors that influence its daily oviposition pattern is crucial if field interventions targeting gravid females are to be successful. This laboratory study investigated the effect of oviposition substrate and time of blood feeding on daily oviposition patterns of *An. gambiae *mosquitoes.

**Methods:**

Greenhouse-reared gravid and hypergravid (delayed oviposition onset) *An. gambiae sensu stricto *and wild-caught *An. gambiae sensu lato *were exposed to three types of substrates in choice and no-choice cage bioassays: water from a predominantly anopheline colonised ground pool (anopheline habitat water), swamp water mainly colonised by culicine larvae (culicine habitat water) and distilled water. The daily oviposition pattern and the number of eggs oviposited on each substrate during the entire egg-laying period were determined. The results were subjected to analysis of variance using the General Linear Model (GLM) procedure.

**Results:**

The main oviposition time for greenhouse-reared *An. gambiae s.s. *was between 19:00 and 20:00 hrs, approximately one hour after sunset. Wild-caught gravid *An. gambiae s.l. *displayed two distinct peak oviposition times between 19:00 and 20:00 hrs and between 22:00 and 23:00 hrs, respectively. During these times, both greenhouse-reared and wild-caught mosquitoes significantly (*P *< 0.05) preferred anopheline habitat water to the culicine one. Peak oviposition activity was not delayed when the mosquitoes were exposed to the less preferred oviposition substrate (culicine habitat water). However, culicine water influenced negatively (*P *< 0.05) not only the number of eggs oviposited by the mosquitoes during peak oviposition time but also the overall number of gravid mosquitoes that laid their eggs on it. The differences in mosquito feeding times did not affect the daily oviposition patterns displayed.

**Conclusion:**

This study shows that the peak oviposition time of *An. gambiae s.l. *may be regulated by the light-dark cycle rather than oviposition habitat characteristics or feeding times. However, the number of eggs laid by the female mosquito during the peak oviposition time is affected by the suitability of the habitat.

## Background

Although *An. gambiae s.l. *mosquitoes are nocturnal in their feeding and oviposition activities, the probable time of oviposition is determined by many factors including ambient temperature and light conditions, and the time the mosquito obtains its blood meal [1,2]. In addition, we hypothesised that the availability of a suitable larval habitat would also affect the mosquito's predisposition to oviposit. *Anopheles gambiae *is discriminative in its oviposition behaviour [3]. Its preferred larval habitats are fresh water pools that are generally small, transient and sunlit, devoid of vegetation and often turbid [4-6]. Oviposition tendency might therefore be related to location and availability of such sites. In this study, we compared the daily oviposition patterns and the number of eggs laid by *An. gambiae s.s. *and wild-caught *An. gambiae s.l. *on aqueous collections from habitats colonised by anopheline or culicine larvae respectively, and distilled water.

## Methods

### Mosquitoes

*Anopheles gambiae s.s. *(MBITA strain; colonised since February 2001) mosquitoes from Mbita Point, western Kenya, were reared in a greenhouse [7] in water obtained from a natural ground pool colonised by anopheline larvae. Average temperatures and relative humidities were 31°C, 52 % during the day and 24°C, 72% at night. The mosquitoes were exposed to the natural photoperiod, 00° 25' South of the equator. A data logger (HOBO™) was used to record ambient conditions. Larvae were fed on Tetramin^® ^fish food. Adult mosquitoes were kept in standard mosquito rearing cages (30 × 30 × 30 cm) made of a metal wire frame with a solid metal base and covered with white nylon mosquito netting. They were offered a 6% glucose solution soaked in white paper towel wicks. Three-to-four-day-old females were offered two blood meals, one each day at 18.00 hrs, from the forearm of a human volunteer. The unfed mosquitoes were removed from the cage after each blood meal. Fully engorged females were left in the cages until they were gravid or hypergravid. Gravid mosquitoes are those that were provided with oviposition substrates on the third evening after their first blood meal. Hypergravid mosquitoes were provided with oviposition substrates one day later. Wild, indoor-resting, blood fed anopheline mosquitoes were collected during early morning hours from houses in Lwanda village of Suba district, western Kenya, by means of aspirators. They were immediately transported to the greenhouse, sorted out to obtain *An. gambiae s.l. *females and provided with 6% glucose solution. They were used in periodicity experiments on the second evening after collection, as described below.

### Oviposition substrates

Turbid water taken from a natural ground pool colonised by anopheline larvae (anopheline habitat water), yellow-brown water from a reed swamp colonised by culicine larvae (culicine habitat water), and distilled water were used as oviposition substrates. Presence of larvae was determined by making five random dips using a 350 ml standard dipper.

### Oviposition substrate preference

The experiments were carried out under greenhouse conditions in 25 cm cubic Plexi^®^-glass cages, each fitted with a white netting top and a side sleeve opening. To determine oviposition substrate preference, individual gravid *An. gambiae s.s. *mosquitoes were exposed to 20 ml of each of the above substrates in a three-choice bioassay (n = 55). The substrates were held in black plastic oviposition cups (2 cm depth, 4 cm diameter), placed at equal distances from one another. Individual mosquitoes were released into the cages at about 17.00 hours and left overnight. The following morning, eggs oviposited on each substrate were counted under a dissection microscope. In subsequent replications, oviposition cups containing substrates were rotated such that they occupied different positions every time in the oviposition cages.

### Daily oviposition patterns in a no-choice bioassay

Daily oviposition patterns of *An. gambiae *female mosquitoes on test oviposition substrates, which were offered individually, were determined as follows. Groups of five greenhouse-reared gravid and hypergravid *An. gambiae s.s. *females were held in separate cages into which anopheline or culicine habitat water or distilled water were introduced. Each mosquito and substrate combination treatment was replicated four times on each experimental day and the experiment repeated on three different days. At the end of the experiment, the mosquitoes that had laid in each group were identified by dissecting each under a dissection microscope and examining their ovaries for the presence of either retained eggs, coiled or uncoiled tracheolar skeins [8].

### Daily oviposition patterns in a choice bioassay

Groups of five gravid and hypergravid *An. gambiae s.s. *(ten cages of each) were placed in separate cages and allowed to choose from the three types of oviposition substrates. Similarly, groups of five wild-caught *An. gambiae s.l. *mosquitoes were offered a choice of the three substrates and their daily oviposition patterns monitored. The experiment was replicated twice on each experimental day and repeated on five different days with new mosquito batches. Individual species within the wild-caught *An. gambiae *mosquitoes that had laid were identified using polymerase chain reaction (PCR) [9].

### Effect of the time of blood feeding on daily oviposition patterns

The effect of the time of blood feeding of *An. gambiae s.s. *on its daily oviposition pattern was determined as follows. Four groups of three-to-four-day-old females were given two blood meals, one each day at 06.00 hrs, 18.00 hrs, 22.00 hrs or at 00.00 hrs, respectively. Unfed females were removed from the cages after every blood meal. Gravid mosquitoes were then provided with oviposition cups on the third day at 06.00 hrs and their daily oviposition patterns monitored.

In all experiments, the oviposition cups were removed from the cages after every hourly interval, for 24 hours, starting at 18.00 hrs and replaced with freshly prepared ones. The eggs laid on each substrate were counted under a dissection microscope. To minimise disturbance that might have been due to exposure to white light, red light was used at night while replacing the oviposition cups.

### Data analysis

Since oviposition trends for gravid and hypergravid females were similar, data for the two were pooled for analysis. The differences in the number of eggs laid on different oviposition substrates were compared statistically by analysis of variance using the General Linear Model (GLM) procedure. The effect of oviposition substrate on the number of either gravid or hypergravid mosquitoes contributing to the total egg number was similarly compared. Means were separated by the least significant difference (LSD) procedure. Data were subjected to log_10 _(n+1) transformation to normalise their distribution. All the analyses were carried out using the SPSS^® ^statistical package, version 11.0.

## Results

### Oviposition substrate preference

The mean number ± standard error (39.4 ± 6.1) of eggs oviposited on anopheline habitat water was significantly higher than that on the culicine (16.1 ± 4.6; *P *= 0.01) or distilled water (23.7 ± 5.3; *P *= 0.02).

### Daily oviposition patterns

**Figure 1:**
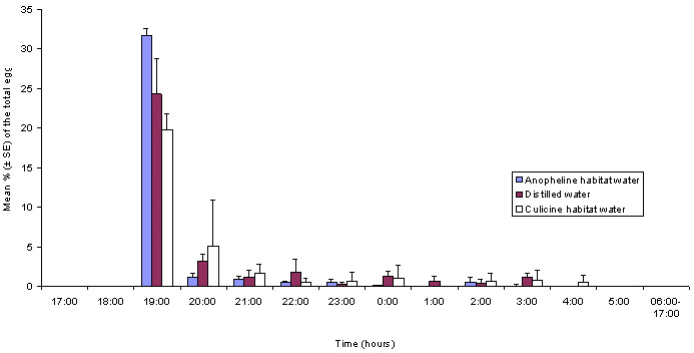
**Daily oviposition patterns of *Anopheles gambiae s.s. *on different oviposition substrates in a no-choice bioassay**. Mean percentage (± SE) of the total eggs laid on each of three different oviposition substrates during 1-h time intervals. n = 24 cages containing five females each. Mosquitoes in each cage were exposed to one type of substrate under a natural LD cycle (sunset at 18:00).

**Figure 2:**
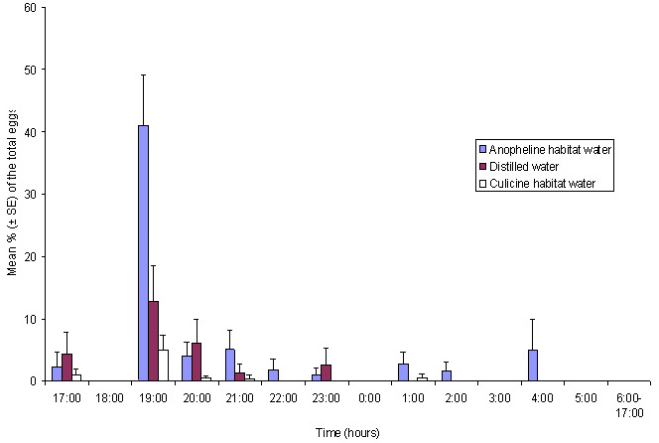
**Daily oviposition patterns of *Anopheles gambiae s.s. *on different oviposition substrates in a choice bioassay**. Mean percentage (± SE) of the total eggs laid on each of the three different oviposition substrates during 1-h time intervals. n = 20 cages containing five females each. Mosquitoes could choose from different substrates placed in the same cage under a natural LD cycle (sunset at 18:00).

Daily oviposition patterns of *An. gambiae s.s. *on different substrates, offered in either no-choice or choice assays, are presented in Figures 1 and 2, respectively. In both cases, the main oviposition time was between 19:00 and 20:00 hrs, approximately one hour after sunset, followed by a steady reduction in the number of eggs laid as the night progressed. In the choice bioassays, the gravid mosquitoes showed significant preference for anopheline habitat water over distilled (*P *= 0.004) or culicine habitat water (*P *= 0.001) throughout the daily cycle. In the no-choice bioassay, although the total number of eggs laid throughout the cycle on the different substrates was different, this was not statistically significant (*P *= 0.4). However, during the peak oviposition time, the eggs laid on anopheline habitat water were significantly more than those on the culicine one (*P *= 0.01) but not significantly more than those on distilled water (*P *= 0.07). Egg-laying by mosquitoes of different ovary development stages was influenced considerably by the type of oviposition substrate (*P *= 0.02). The hypergravid/ anopheline habitat water combination had the highest average number of mosquitoes (4.4 ± 0.3) laying their eggs, whereas gravid/culicine combination yielded the lowest response (2.5 ± 0.4; Table 1).

**Table 1:** The number of mosquitoes (Mean ± SE^1^) contributing to the total eggs laid in each mosquito/ substrate combination.

**Mosquito/ Substrate**	**Mean ± SE^1^**
Gravid/ Distilled water	3.3 ± 0.4^bc^
Gravid/ Anopheline habitat water	3.5 ± 0.4^ab^
Gravid/ Culicine habitat water	2.5 ± 0.4^c^
Hypergravid/ Distilled water	3.8 ± 0.4^ab^
Hypergravid/ Anopheline habitat water	4.4 ± 0.3^a^
Hypergravid/ Culicine habitat water	3.6 ± 0.4^ab^

^1^SE: Standard Error. n = 12 cages each containing five mosquitoes. Any two means sharing a letter in common are not significantly different at 5% level (LSD test).

**Figure 3:**
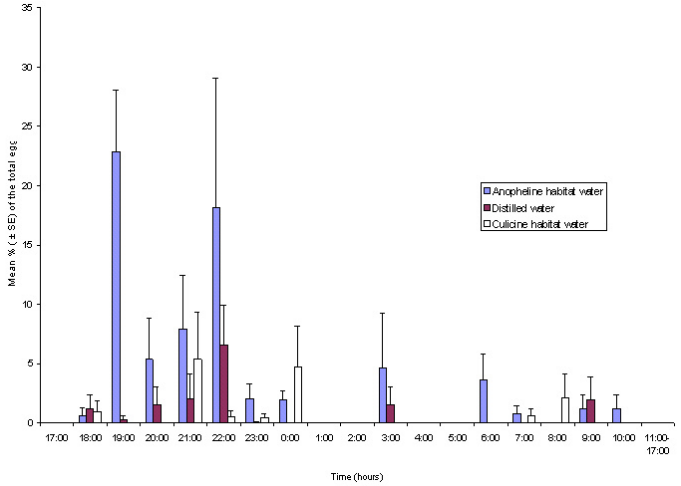
**Daily oviposition patterns of wild-caught *Anopheles gambiae s.l. *on different oviposition substrates in a choice bioassay**. Mean percentages (± SE) of the total eggs oviposited on each of the three different oviposition substrate during 1-h time intervals. n = 10 cages containing five females each. Mosquitoes could choose from different substrates placed in the same cage under a natural LD cycle (sunset at 18:00).

Unlike the greenhouse-reared *An. gambiae s.s.*, the wild-caught *An. gambiae s.l.*, which consisted of 23.9% *An. gambiae s.s.*,71.7% *An. arabiensis *and 4.4% unidentified gravid females (n = 46), displayed two main oviposition times early in the night, between 19:00 and 20:00 hrs and between 22:00 and 23:00 hrs, respectively (Figure 3). These mosquitoes also showed significant preference (*P *= 0.01) for anopheline habitat water over distilled or culicine habitat water.

**Figure 4:**
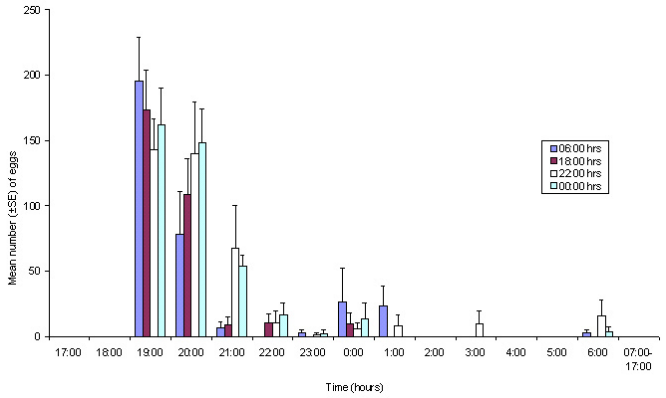
**Daily oviposition patterns of *Anopheles gambiae s.s. *fed at different times**. Mean number (± SE) of eggs oviposited during 1-h time intervals. n = 8 cages containing five females each. Mosquitoes were kept under a natural LD cycle (sunset at 18:00).

*An. gambiae s.s. *females that obtained their blood meals later in the night displayed a somewhat broader oviposition peak time interval, ranging from 19:00 hrs to 22:00 hrs (Figure 4), than those that had fed earlier on, whose peak oviposition time interval was narrower (19:00 hrs to 21:00 hrs).

## Discussion

In the present study, the daily oviposition patterns of greenhouse-reared *An. gambiae s.s. *were well defined with oviposition peak times between 19:00 and 20:00 hrs, regardless of the type of oviposition substrate used. Haddow and Ssenkubuge [10] obtained comparable results using *An. gambiae s.s. *(KISUMU strain, western Kenya): about half of the eggs were laid during the first three hours of the night (18:00 – 21:00 hrs). On the other hand, oviposition by wild-caught mosquitoes from the coast of Kenya used by McCrae [1], comprising mostly *An. gambiae s.s.*, peaked much later at night in the hour following midnight. This suggests differences in oviposition patterns between our strain and that of Haddow and Ssenkubuge representing Lake Victoria populations, on one hand, and that used by McCrae representing the Kenyan coastal population, on the other. Studies of oviposition patterns of populations from different parts of eastern Africa may help shed further light on the question.

In the current study, wild-caught *An. gambiae s.l.*, which were shown to contain a mixture of *An. gambiae s.s. *and *An. arabiensis *gravid females, displayed two distinct oviposition peak times in the first half of the night. The two peaks may be attributed to the two sibling species and suggests that this may also be an important factor in the diversity of oviposition patterns in the field in different geographical locations.

The differences in the mosquito feeding times did not affect the timing of peak oviposition, although females that obtained their blood meals later in the night displayed a somewhat broader oviposition peak interval. Peak oviposition consistently occurred approximately one hour after sunset; therefore, a fall in light intensity might be one of the important cues that trigger oviposition in female *An. gambiae *that are physiologically ready to oviposit. On the other hand, McCrae [1] observed that the time of oviposition was a function of the time of blood feeding and not a result of an endogenous rhythm. Given the uniform oviposition peak times of mosquitoes that were fed at different times, daily oviposition among *An. gambiae s.l. *may also be endogenously regulated. Detailed experiments to demonstrate a free-running oviposition periodicity would clarify this. There was no difference in oviposition patterns displayed by gravid and hypergravid mosquitoes. Since significantly more gravid females exposed to the preferred substrate oviposited their eggs than those exposed to the less preferred one, gravid females that fail to find a suitable oviposition site on the night they are due may retain their eggs and oviposit early the next night as hypergravids.

Gravid mosquitoes are generally attracted to water; however, the decision to oviposit may depend on additional olfactory signals [11] and /or contact stimuli received when the insects land on the water surface [12]. In this study and others [13], the gravid mosquitoes showed marked preference for the water taken from a site naturally inhabited by anopheline larval populations. This suggests 'memory' of similar information gathered by contact with the oviposition water at emergence or during larval period as in the case of *Culex quinquefasciatus *[14]. In this regard, gravid females might associate specific site characteristics from conspecific and heterospecific immatures, soil microbial activity [11], colour and turbidity of the oviposition substrate [13] with their suitability for sustaining progeny development.

## Conclusions

This study shows that the peak oviposition time of *An. gambiae s.l. *may be regulated by the light-dark cycle rather than oviposition habitat characteristics or feeding times. However, the number of eggs laid during the peak oviposition time is affected by the suitability of the habitat. This suggests that there is a relationship between the investment made by the female mosquito with respect to the number of eggs laid in a given habitat and the potential fitness of the progeny. Females may use a series of site characteristics, including olfactory cues, to locate and oviposit at such sites. Our results on oviposition patterns differ from those reported on a coastal population, and suggest that a lot more work needs to be done to elucidate differences in this regard between different populations.

## Competing interests

The authors declare that they have no competing interests.

## Authors' contributions

LAS and KO conducted all the experimental work. AH, ALD, BGJK, JCB and JG co-ordinated and/or supervised the work. All authors actively contributed to the interpretation of the findings and development of the final manuscript and approved the final manuscript.
